# Diplopia from abducens nerve paresis as a presenting symptom of COVID-19: a case report and review of literature

**DOI:** 10.5935/0004-2749.20220028

**Published:** 2025-08-21

**Authors:** Geulah S. Ben-David, Orly Halachmi-Eyal, Hana Shyriaiev, Shay Brikman, Guy Dori, Daniel Briscoe

**Affiliations:** 1 Department of Ophthalmology, Emek Medical Center, Afula, Israel; 2 Ruth and Bruce Rappaport Faculty of Medicine, Technion-Israel Institute of Technology, Haifa, Israel; 3 Department of Internal Medicine E, Emek Medical Center, Afula, Israel

**Keywords:** Diplopia, Severe Acute Respiratory Syndrome, Ophthalmoplegia, Coronavirus infection, Abducens nerve disease, SARS-CoV-2, Pandemic, Humans, Case report, Diplopia, Síndrome Respiratória Aguda Grave, Oftalmoplegia, Infecção por coronavírus, Doença do nervo abducente, SARS-CoV-2, Pandemia, Humanos, Relato de caso

## Abstract

Neurological manifestations of novel coronavirus disease 3019 (COVID-19) remain unclear. We report the case of a 44-year-old febrile man who presented with double vision and headache 2 d after initial symptoms of fatigue, generalized muscle weakness, and loss of appetite. He was subsequently diagnosed with COVID-19 and transient abducens nerve paresis. He did not present with any respiratory symptoms or additional specific neurological findings. We recommend that with the rising number of cases across the world, physicians develop a greater index of suspicion for COVID-19 in patients with cranial neuropathies, even in those with mild disease without typical respiratory symptoms.

## Abstract (pt)

As manifestações neurológicas de COVID-19 não são bem compreendidas. Relatamos o caso de um homem febril de 44 anos que apresentou visão dupla e dor de cabeça dois dias após os sintomas iniciais de fadiga, fraqueza muscular generalizada e perda de apetite. O paciente foi posteriormente diagnosticado com COVID-19 e paresia transitória do nervo abducente, não apresentando sintomas respiratórios ou achados neurológicos específicos adicionais. Como o número de casos tem aumentado em todo o mundo, sugerimos que os médicos tenham um índice de suspeita maior para COVID-19 em pacientes com neuropatias cranianas, mesmo em casos leves sem sintomas respiratórios típicos.

## INTRODUCTION

The novel coronavirus disease (COVID-19) that originated in Wuhan, China has become a widespread global pandemic with over 16 million cases and over 650,000 deaths worldwide^(1)^. Linked to the family of severe acute respiratory syndrome coronavirus (SARS-CoV), the new coronavirus is designated as SARS-CoV-2 with typical symptoms, including fever, cough, and shortness of breath^(1)^. The main reported ocular abnormality is conjunctivitis. Recently, in a study on 38 patients with confirmed COVID-19, 12 (31.6%) had conjunctivitis^(2)^. Neurological manifestations have been reported in 78/214 (36.4%) of COVID-19 patients, including central nervous system (e.g., headache) and peripheral nervous system (e.g., taste, smell, and vision impairment) manifestations^(3)^. Diplopia secondary to abducens nerve palsy in adults is often secondary to vascular disease, trauma, tumors, autoimmune, inflammatory, and idiopathic causes. Spontaneous recovery may occur in unilateral, isolated, and benign cases^(4)^. Diplopia secondary to cranial nerve motor neuropathies may be associated with viral etiology; however, it is not a common presenting symptom of COVID-19. Awareness of cranial nerve involvement may increase the index of suspicion for diagnosing patients with COVID-19 and potentially help in preventing disease transmission. Here, we describe the first case of isolated abducens paresis and associated diplopia as the sole ocular abnormality in a generally healthy COVID-19 patient.

The data in the current case report were obtained from hospital medical records. Informed consent was obtained from the patient for the publication of this case report. The literature search included a systematic review on the PUBMED databases based on the combination of search terms “diplopia” OR “ophthalmoparesis” AND “SARS-CoV-2” OR “COVID-19”. Case reports, case series, editorials, reviews, case-control studies, and cohort studies were evaluated for their relevance to the current investigation, and relevant references were screened and included. Two authors (HS and GBD) conducted the literature search until August 18, 2020.

## CASE REPORT

**Figure 1:**
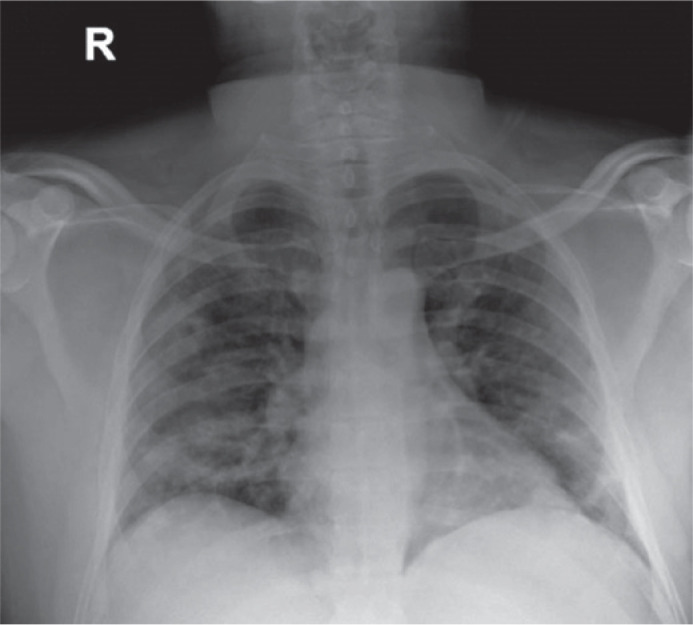
Chest radiograph of a patient with abducens nerve palsy and COVID-19. Imaging evaluation of a 44-year-old man with COVID-19 reveals bilateral interstitial opacities indicating a pneumonia-like illness that did not manifest with any symptoms.

**Figure 2:**
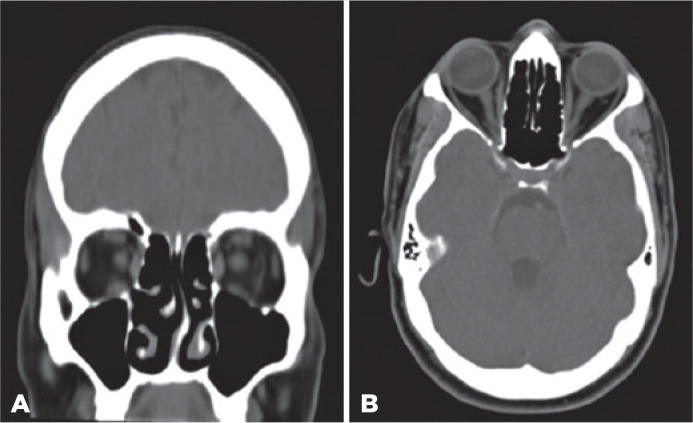
Head CT scan without contrast. Neuroimaging evaluation in a 44-year-old man with COVID-19 infection reveals no evidence of cranial mass lesions, intra-cerebral hemorrhage, ischemia, or other pathological findings viewed with (A) coronal and (B) axial section scans.

A 44-year-old generally healthy man who was referred for ophthalmologic consultation in a hospitalized institutional setting presented with a 5-day history of fever, double vision, and headache. The patient also reported fatigue, generalized muscle weakness, and loss of appetite for 1 wk. Several days before his admission, he had visited a healthcare practitioner and was sent home with antipyretic treatment. Owing to the absence of respiratory symptoms, COVID-19 was not suspected at that time. The patient had no known medical or ocular history, medications, or allergies. As his symptoms did not resolve, he was admitted to the hospital without any respiratory symptoms. Blood tests demonstrated mild lymphopenia (1.28 × 10^9^/L), elevated C-reactive protein (92 mg/L), and D-dimer level (1.3 mg/mL) consistent with COVID-19. Lung auscultation and chest radiography indicated a bilateral pneumonia-like illness (Figure 1) that was asymptomatic. Computed tomography of the head showed no pathological findings (Figure 2). Reverse transcriptase-polymerase chain reaction (RT-PCR) was positive for SARS-CoV-2 in the patient’s first nasopharyngeal swab. Ophthalmologic examination, performed with complete personal protective equipment, revealed binocular diplopia and a limitation to abduction in the left eye. Orthophoria was noted in the right gaze. There was no additional cranial nerve involvement. Ophthalmologic examination, including pupillary response, anterior pole, and dilated fundus exam was otherwise unremarkable. Neurological examination showed normal results without any evidence of meningeal irritation. Lumbar puncture and magnetic resonance imaging were considered but not performed because symptoms resolved within 5 d of admission. Treatment included azithromycin and hydroxychloroquine for 5 d, as per the local infectious disease protocol. The patient was discharged to state-mandatory quarantine; 10 d thereafter, his condition had improved, and he tested negative for SARS-CoV-2.

## DISCUSSION

The most plausible diagnosis in our patient who had no prior medical or ocular history was isolated abducens paresis associated with COVID-19 infection. Vascular, neoplastic, and traumatic etiologies as well as pre-existing strabismus, thyroid eye disease, and myasthenia gravis were ruled out. The number of recognized neurologic manifestations of infection is rapidly increasing. These may result from a variety of mechanisms, including virus-induced hyperinflammatory and hypercoagulable states, direct virus infection of the CNS, and post-infectious immune-mediated processes. The elevated D-dimer level in our patient may be consistent with micro-angiopathy and hypercoagulability mechanisms. Although our patient was otherwise healthy, an ischemic episode in the vaso nervorum causing temporary abducens nerve palsy may also be considered. Prior CoV studies have reported viral spread via the olfactory nerves, possibly due to a viral interaction with the membrane bound angiotensin-converting enzyme 2 receptor^(5)^. Another hypothesized mechanism is hyperactivation of monocytes and dysregulated macrophages leading to a hyperinflammatory immune response^(6)^. Diplopia occurred at the beginning of our patient’s disease course; therefore, the precise mechanism of cranial nerve involvement remains unknown.

Recently, diplopia has been suggested as a symptom of COVID-19 linked to ophthalmoparesis and Miller Fisher Syndrome, a demyelinating inflammatory polyneuropathy. In 3 recent studies, one patient presented with partial third nerve palsy and accompanying bilateral sixth nerve palsy, one with complete third nerve palsy only, one with bilateral sixth nerve palsy, and two with unilateral sixth nerve palsy^(7-9)^. A review article investigating the neurologic implications in COVID-19 presented the case of a patient with facial nerve palsy during hospitalization with confirmed SARS-CoV-2 infection^(10)^. Similar to that in the above-mentioned cases, the precise mechanism in our case remains unknown. In these previously mentioned studies, patients who presented with third nerve palsy with or without additional sixth nerve palsy had more severe manifestations of their infection and required more intensive, in-patient treatment. It remains unclear whether we can provide a clinical guideline for future cases. However, the presentation and management of isolated ocular motor cranial nerve paresis, as in our case, may be associated with a less complicated disease process, while patients who present with persistent sixth nerve palsy or additional third nerve palsy may have greater disease severity.

The presence of lymphopenia and additional neurological signs and symptoms in this case, such as fatigue, muscle weakness, and loss of appetite are characteristic of COVID-19. The absence of severe neurological deficits, such as stroke and impaired consciousness, suggests that his disease course was mild to moderate with very limited neurological involvement. Chest computed tomography, magnetic resonance neuroimaging, and cerebral spinal fluid analyses were not performed for our patient who presented during the initial peak of the coronavirus pandemic; thus, our conclusions are limited with respect to the precise mechanism of our patient’s disease. Treatment with azithromycin and hydroxychloroquine for 5 d as per the institutional protocol coincided with patient improvement and symptom cessation. In conclusion, here we describe the case of a patient with unilateral abducens paresis and resulting diplopia as the sole ocular abnormality in a generally healthy male with mild COVID-19. As cases continue to rise, physicians should be aware that patients with COVID-19 may present with cranial nerve involvement even if the disease severity is mild and there are no typical respiratory symptoms.
